# Characterization and Neural Modeling of a Microwave Gas Sensor for Oxygen Detection Aimed at Healthcare Applications †

**DOI:** 10.3390/s20247150

**Published:** 2020-12-13

**Authors:** Zlatica Marinković, Giovanni Gugliandolo, Mariangela Latino, Giuseppe Campobello, Giovanni Crupi, Nicola Donato

**Affiliations:** 1 Faculty of Electronic Engineering, University of Niš, Aleksandra Medvedeva 14, 18000 Niš, Serbia; zlatica.marinkovic@elfak.ni.ac.rs; 2 MIFT Department, University of Messina, Viale F. Stagno d’Alcontres 31, 98166 Messina, Italy; giovanni.gugliandolo@unime.it (G.G.); mlatino@unime.it (M.L.); 3 Department of Engineering, University of Messina, Contrada di Dio, S. Agata, 98166 Messina, Italy; gcampobello@unime.it (G.C.); ndonato@unime.it (N.D.); 4 BIOMORF Department, University of Messina, Via Consolare Valeria, 98100 Messina, Italy

**Keywords:** artificial neural networks, bioengineering, healthcare applications, interdigital capacitor, oxygen sensing, scattering parameter measurements

## Abstract

The studied sensor consists of a microstrip interdigital capacitor covered by a gas sensing layer made of titanium dioxide (TiO_2_). To explore the gas sensing properties of the developed sensor, oxygen detection is considered as a case study. The sensor is electrically characterized using the complex scattering parameters measured with a vector network analyzer (VNA). The experimental investigation is performed over a frequency range of 1.5 GHz to 2.9 GHz by placing the sensor inside a polytetrafluoroethylene (PTFE) test chamber with a binary gas mixture composed of oxygen and nitrogen. The frequency-dependent response of the sensor is investigated in detail and further modelled using an artificial neural network (ANN) approach. The proposed modelling procedure allows mimicking the measured sensor performance over the whole range of oxygen concentration, going from 0% to 100%, and predicting the behavior of the resonant frequencies that can be used as sensing parameters.

## 1. Introduction

The recent development of the ambient-assisted living concept and the extraordinary progress of bioengineering and healthcare applications have enforced the development of a wide range of sensors aimed at sensing different environmental and biomedical parameters [1,2,3,4,5]. Sensing gas presence is very important not only for ensuring a safe living environment without dangerous gases, but also for being of help in the diagnosis of certain illnesses, such as diabetes, cancer, etc. [6,7,8,9,10,11,12,13], as well as in therapeutic applications [14,15]. In recent years, various sensors based on microwave devices have been developed to be used for gas sensing purposes [16,17,18,19,20,21,22,23,24,25,26,27,28,29,30,31,32,33,34,35,36,37,38]. Compared to their conventional counterparts based on resistive, capacitive, and amperometric effects [18], sensors based on microwave transducers show better performance, i.e., they have lower power consumption, a shorter response time, and a lower operating temperature [16,18,19,20,21,22]. A low-cost electronic interface can be developed for such sensors with an accurate resonant frequency estimation [39,40,41,42]. Moreover, they can be easily integrated into antennas, thereby being compatible with wireless technologies [25,26,43]. It is worthy of note that, through integration with microfluidic channels, microwave sensors can also allow dielectric characterization of biological liquids [44,45,46,47,48]. In recent years, many efforts have been devoted to the development of the materials, design, and applications of miniature devices for gas sensing [49,50]. Among the many applications, gas sensors used for oxygen detection are particularly notable because of their use in monitoring pollution emissions from various sources (e.g., motor vehicles and industrial combustions) [50,51].

**Figure 1:**
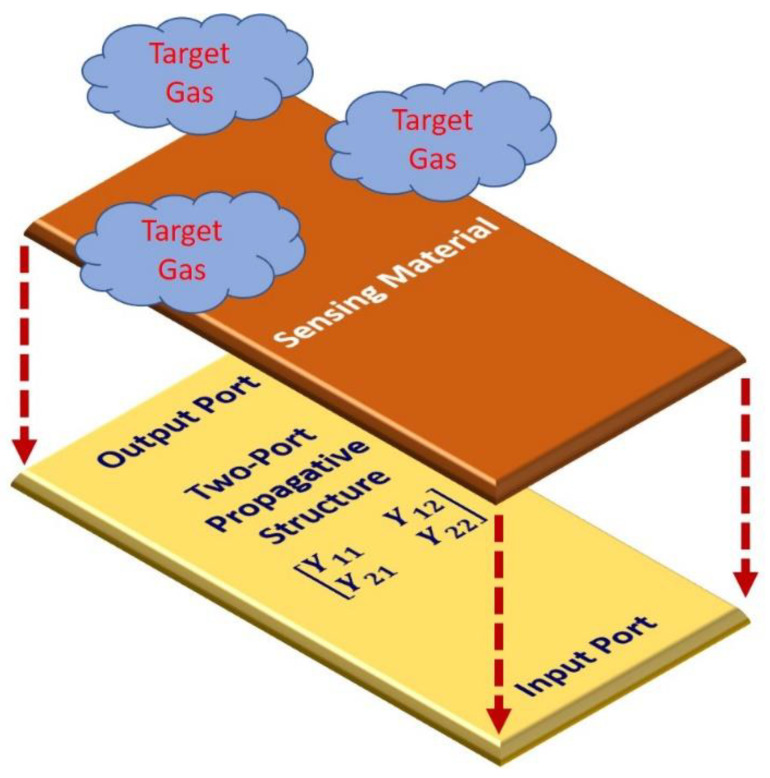
Illustration of the working principle of a gas sensor based on using a microwave device as a propagative structure and a sensing layer for gas detection. The two-port gas sensor is obtained by deposition of the gas sensing layer on the propagative structure and then exposure to the target gas.

A microwave transducer consists of a microwave device, acting as an electromagnetic wave propagative structure, which is covered with a sensing layer, as illustrated in Figure 1. When exposed to the target gas, the sensing layer material interacts with the gas molecules and, consequently, the dielectric properties and the device electrical response are changed. Therefore, the changes in the target gas concentration influence the changes in the electrical response of the sensor. Often, the propagative structure is realized in the microstrip technology, such as microstrip patch antennas [24,25,26], microwave resonators [20,27,28,29,30,31,32], and other microstrip structures [35,36]. Among the various exploited structures, sensors based on the use of interdigital capacitors (IDCs) as propagative structures have been proposed [30,37]. The gas sensor considered in this work is a two-port IDC fabricated in microstrip technology and covered with a thick layer of titania (TiO_2_), as a sensitive layer. TiO_2_ is widely known in scientific literature as a sensing material, such as the resistive gas sensors developed by employing Pt-TiO_2_/MWCNTs (multi-walled carbon nanotubes) hybrid composites [52] and TiO_2_ coated carbon nanotubes prepared by atomic layer deposition (ALD) [53]. Furthermore, the sensing properties and photochromism of Ag-TiO_2_ nanoheterostructures were also evaluated [54]. The TiO_2_ metal oxide is suitable for the present purpose since the O_2_ molecules absorbed on its surface can be easily desorbed, even at room temperature [33,54]. The developed prototype is used as a probe for oxygen (O_2_) sensing. A possible application for this device is in oxygen therapy, where the O_2_ concentration is usually greater than 85%. The device could be also useful in spacecraft oxygen level detection; in these cases, the O_2_ concentration is above 60% [55].

The main aim of this paper is to develop and exploit a model able to mimic accurately the electrical response in a frequency range spanning from 1.5 GHz to 2.9 GHz, and for different concentrations of O_2_, ranging from 0% to 100% *v*/*v* at a room temperature. To develop the model, artificial neural networks (ANNs) have been chosen as the modelling tool. Owing to their ability to learn the relationship between an input–output set of data, ANNs have found a variety of applications in different research fields, such as microwave device modelling [56,57,58,59,60,61,62,63,64,65,66] and gas sensing purposes [67,68,69,70,71,72,73,74,75]. As far as microwave device modelling is concerned, the ANNs have often been applied to model the device’s electrical characteristics versus different operating and ambient conditions, as well as versus device dimensions making the model scalable. In the gas sensing applications, the ANNs are, typically, exploited for gas concentration sensing, gas classification, and modelling of the gas sensor characteristics. In the present case, an ANN model is developed to reproduce faithfully the device’s electrical characteristics. The admittance (*Y*−) parameter representation is adopted, and particular attention is given to the shift in the resonant frequencies of *Y*_11_, which can be used as sensing parameters for detecting the concentration of the gas that the sensor is exposed to. To model the sensor behavior under different gas concentrations, the ANNs are exploited. Namely, the developed model relates the *Y*_11_ real and imaginary parts of the sensor with the frequency and O_2_ concentration by using a two-step hierarchical prior-knowledge-input (PKI) neural approach. This study extends the previous findings reported in [37,38], focusing on a thorough investigation of the neural modelling approach for mimicking the shift in the resonant frequencies with the changes of the gas concentration variations, as well as their sensitivity-based analysis. The learning and generalization capabilities of the neural-based approach are exploited for modelling the resonant frequencies, in order to strengthen their possible usage as gas concentration sensing markers.

The paper is organized as follows. The studied sensor, experimental set-up, and a brief analysis of the measured electrical characteristics are given in Section 2. Section 3 contains a description of the developed ANN-based model. The most illustrative results, followed by a corresponding discussion, are presented in Section 4. Finally, Section 5 contains the main concluding remarks.

## 2. Studied Sensor and Experimental Set-up

**Figure 2:**
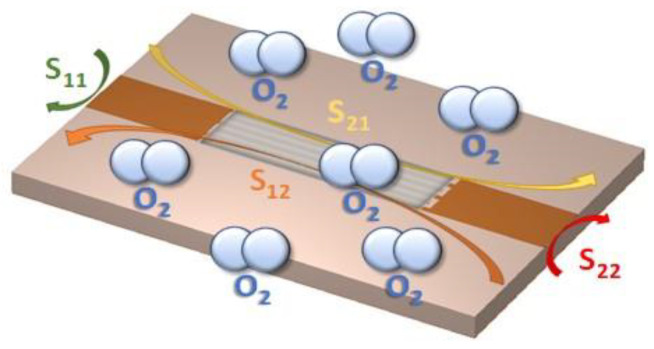
Illustration of the experimental characterization of the developed sensor for oxygen sensing.

**Figure 3:**
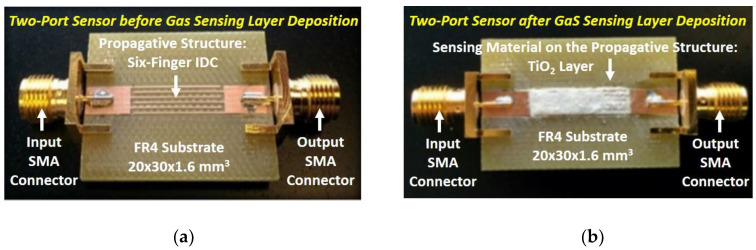
Photos of the sensor: (**a**) before and (**b**) after the gas sensing layer deposition.

As mentioned in the introductory section, a two-port IDC structure covered by a gas sensing layer is employed as a microwave transducer for oxygen sensing (see Figure 2). The studied device is made of six fingers with the following dimensions: a length (*L_f_*) of 15 mm, a width (*W_f_*) of 0.36 mm, and a spacing (*S*) of 0.36 mm. Microstrip lines with the input impedance of 50 Ω are connected at the SMA connectors at the input and output ports of the device. The structure is fabricated on a 1.6-mm FR4 substrate by using the LPKF Protomat S103 PCB milling machine. The dielectric constant (*ε_r_*) and the loss tangent (tan*δ*) of the substrate are 4.2 and 0.015, respectively. The board dimensions are relatively small (i.e., 20 mm × 30 mm × 1.6 mm). A picture of the fabricated device is shown in Figure 3a. As a sensitive layer, a thick layer of TiO_2_ is deposited by screen printing at the top of the fabricated IDC. The employed TiO_2_ is composed of 56.5% anatase, 19.8% rutile, and 23.6% brookite [54]. To reduce the humidity content, the device with the deposited sensing layer is placed inside an oven at 60 °C for two hours. A picture of the device with the deposited sensing layer is reported in Figure 3b.

**Figure 4:**
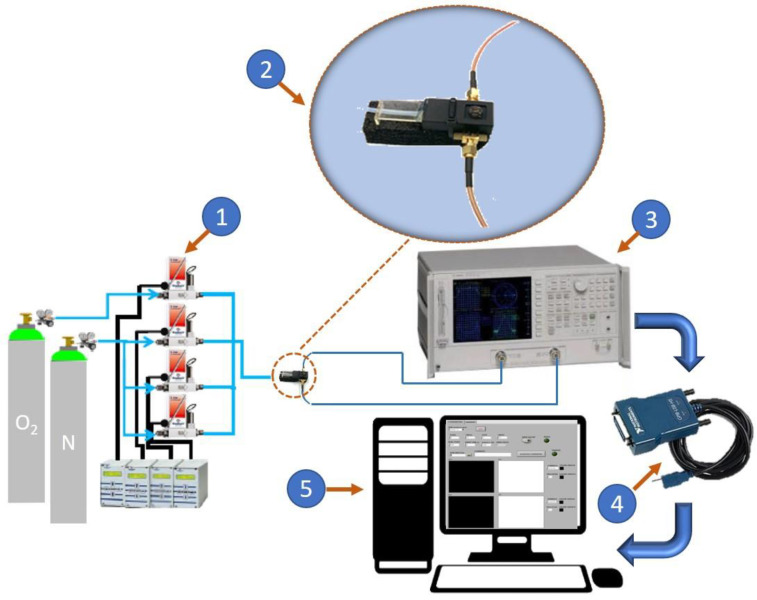
Illustration of the experimental set-up. It consists of five main blocks: (1) the controlled gas system, (2) the measurement chamber, (3) the vector network analyzer, (4) the USB/GPIB interface, and (5) the graphical user interface.

An illustration of the experimental set-up is shown in Figure 4. It is a fully automated gas control system consisting of certified gas bottles and mass flow controllers, in order to set the preferred gas mixture inside the testing chamber and create a controlled atmosphere. Therefore, it is possible to select the gas mixture, set the concentration value for each gas, and control/monitor the gas flow. The sensor under test (SUT), which is being characterized, is placed inside a polytetrafluoroethylene (PTFE) test chamber. The small chamber volume (5 cm^3^) allows for the setting or purging of the gas content in a short time. The device is exposed to a binary gas mixture composed of oxygen and nitrogen (N_2_). The O_2_ concentration is varied from 0% (pure N_2_ inside the chamber) to 100% (no N_2_ inside the chamber). The gas flow, set to 100 cm^3^/min, is maintained constant for the whole measurement session. The SUT is connected to the Agilent 8753ES vector network analyzer (VNA) that is used to perform the frequency-dependent measurements. The measured data are transferred via the USB/GPIB interface to a personal computer, where they are stored. The sensor electrical response is determined by measuring the scattering (*S*−) parameters under different conditions of O_2_ concentration. The S-parameters are complex and frequency-dependent quantities that allow for full characterization of the electrical behaviorbehavior of linear devices. However, as discussed below, instead of the *S*-parameters, it is more suitable to carry out the investigation using the impedance (*Z*−) or admittance (*Y*−) parameters, which are equivalent representations that can be straightforwardly calculated from the measured *S*-parameters by means of the well-known conversion formulas [76,77]. As an illustrative example, we report here the conversion formulas from *S*- and *Z*- parameters to the *Y*-parameters:

(1) $$\begin{array}{l} \left[\begin{array}{c} Y_{11\;\;\;\;}\;\;\;Y_{12}\\ Y_{21\;\;\;\;}\;\;\;Y_{22} \end{array}\right]=\left[\begin{array}{c} {\frac{Z_{22}}{Z_{11}Z_{22-Z_{12}Z_{21}}}}_{\;\;\;\;}\;\frac{-Z_{12}}{Z_{11}Z_{22-Z_{12}Z_{21}}}\\ \frac{-Z_{21}}{Z_{11}Z_{22-Z_{12}Z_{21}}}\;\;\;\;\frac{Z_{11}}{Z_{11}Z_{22-Z_{12}Z_{21}}} \end{array}\right]\\ =\left[\begin{array}{c} Y_{0}{\frac{\left(1-S_{11}\right)\left(1+S_{22}\right)+S_{12}S_{21}}{\left(1+S_{11}\right)\left(1+S_{22}\right)-S_{12}S_{21}}}_{\;\;\;\;}\;\;\;\;-Y_{0}\frac{2S_{12}}{\left(1+S_{11}\right)\left(1+S_{22}\right)-S_{12}S_{21}}\\ -Y_{0}\frac{2S_{21}}{\left(1+S_{11}\right)\left(1+S_{22}\right)-S_{12}S_{21}}\;\;\;\;Y_{0}\frac{\left(1+S_{11}\right)\left(1-S_{22}\right)+S_{12}S_{21}}{\left(1+S_{11}\right)\left(1+S_{22}\right)-S_{12}S_{21}} \end{array}\right] \end{array}$$

where *Y*_0_ is the characteristic admittance (normally 20 mS).

**Figure 5:**
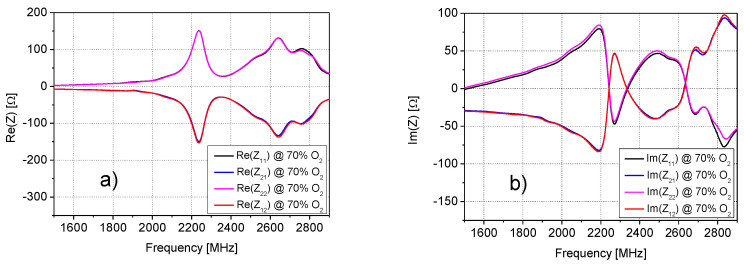
Frequency dependence of the (**a**) real and (**b**) imaginary parts of the impedance parameters at 70% of oxygen (O_2)_ concentration.

**Figure 6:**
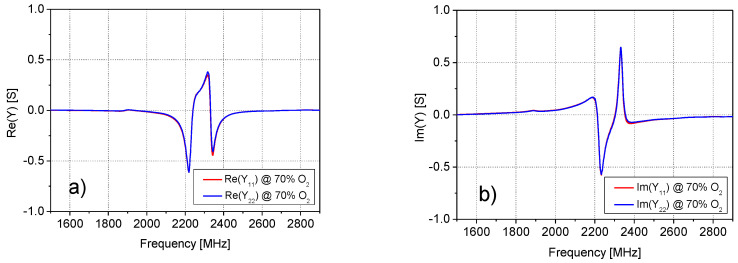
Frequency dependence of the (**a**) real and (**b**) imaginary parts of the admittance parameters *Y*_11_ and *Y*_22_ at 70% of O_2_ concentration.

**Figure 7:**
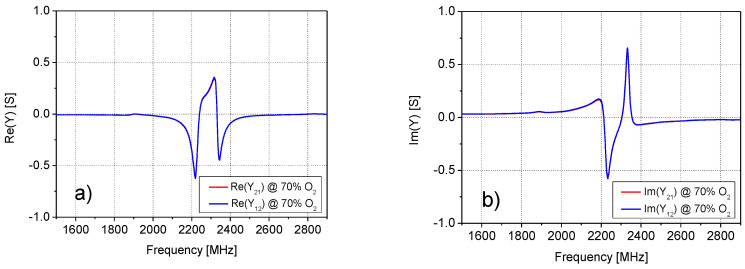
Frequency dependence of the (**a**) real and (**b**) imaginary parts of the admittance parameters *Y*_21_ and *Y*_12_ at 70% of O_2_ concentration.

The analysis of the *Z*− and *Y*− parameters at all studied O_2_ concentrations showed that the impedance parameters of the SUT quite closely satisfy the condition *Z*_11_ = *Z*_22_ = −*Z*_12_ = −*Z*_21_, and, analogously, the admittance parameters are quite close to the condition *Y*_11_ = *Y*_22_ = *Y*_12_ = *Y*_21_. As an illustrative example of the validity of these approximations, Figure 5, Figure 6 and Figure 7 report the frequency dependence of the *Z*− and *Y*− parameters at 70% of O_2_ concentration. Therefore, *Y*_11_ has been selected for further investigation and modelling, since it is enough to study the behavior of only one parameter versus the frequency and O_2_ concentrations. This made the analysis simpler and faster.

**Figure 8:**
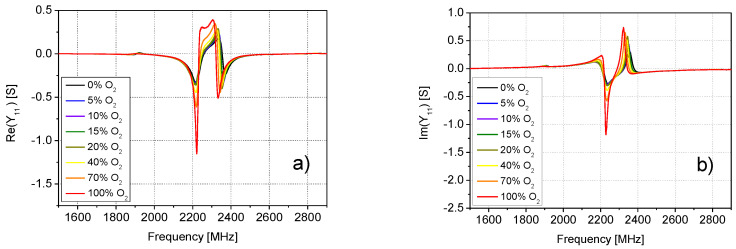
Frequency dependence of the (**a**) real and (**b**) imaginary parts of *Y*_11_ at different O_2_ concentrations.

By analyzing the admittance parameter *Y*_11_ for all considered concentrations of O_2_ (see Figure 8), it was concluded that a substantial variation of the admittance parameter was observed in the frequency range from 2.0 GHz to 2.5 GHz. Particular attention has then been devoted to this frequency range.

## 3. ANN-Based Model

**Figure 9:**
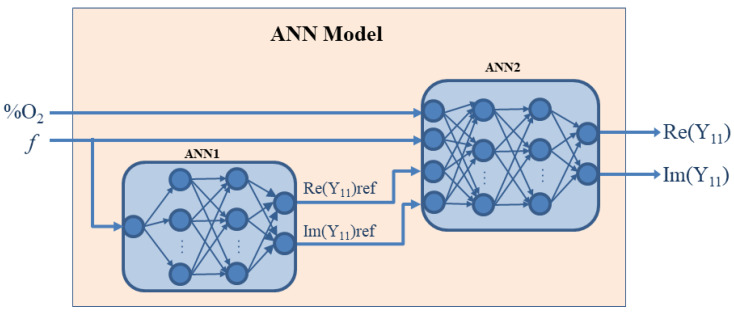
Illustration of the proposed artificial neural network (ANN) model for reproducing the frequency dependence of the measured *Y*_11_ of the studied sensor operating at different concentrations of O_2_.

A modelling approach based on multilayer perceptron ANNs is proposed to model the behavior of the admittance parameters versus the frequency and the O_2_ concentration, as shown in Figure 9 [38]. Since the admittance parameters closely satisfy the condition *Y*_11_ = *Y*_22_ = *Y*_12_ = *Y*_21_, it is enough to develop a model only for the parameter *Y*_11_. For that purpose, an ANN having two outputs referring to the real and imaginary parts of *Y*_11_ should be trained by using the values of the measured *Y*_11_. The ANN has two input neurons corresponding to the O_2_ concentration and frequency (see ANN2 in Figure 9). Moreover, the proposed ANN has, besides the two mentioned inputs, two additional inputs referring to the real and imaginary parts of *Y*_11_ at a reference concentration. Namely, in order to ensure the modelling accuracy, a so-called prior-knowledge-input neural modelling approach is adopted [56]. The PKI approach assumes that the ANN has additional inputs, which are correlated in a certain way to the ANN outputs, with the aim of more easily achieving the desired accuracy with the available set of data. As the prior-knowledge, *Y*_11_ at a chosen reference O_2_ concentration is exploited, since the shape of the frequency-dependent behavior of *Y*_11_ is roughly similar for all concentrations. For determining *Y*_11_ at the chosen reference frequency, an additional ANN is considered, namely ANN1 in Figure 9. It has one input and two output neurons. Both ANNs can have one or two layers of hidden neurons. The number of hidden neurons is determined during the ANN training process, as it is not possible to determine it a priori. Namely, for each ANN, ANN structures with a different number of hidden neurons are trained and then, after comparing their accuracy, the best one is chosen as the final model.

The procedure of the model development is as follows. As the first step, the ANN1 is trained using the values of the measured *Y*_11_ at the chosen reference concentration and in the considered frequency range. As the next step, the training dataset for ANN2 development is built. Namely, the measured frequency-dependent behavior of the real and imaginary parts of *Y*_11_ for different O_2_ concentrations is accompanied by the values of *Y*_11_ at the reference concentration calculated from ANN1 for the same frequency. Once the ANN2 is trained, the two ANNs form the model that can be further used. It is noteworthy that the ANNs can be described with the equivalent mathematical expressions, which can be straightforwardly implemented in a circuit simulator or any math-based environment. In particular, the proposed ANN model can be integrated with a simulator and used for accurate modelling of gas sensors. Such a model might reduce both processing time and analytical complexity, resulting in a simpler computation process.

**Figure 10:**
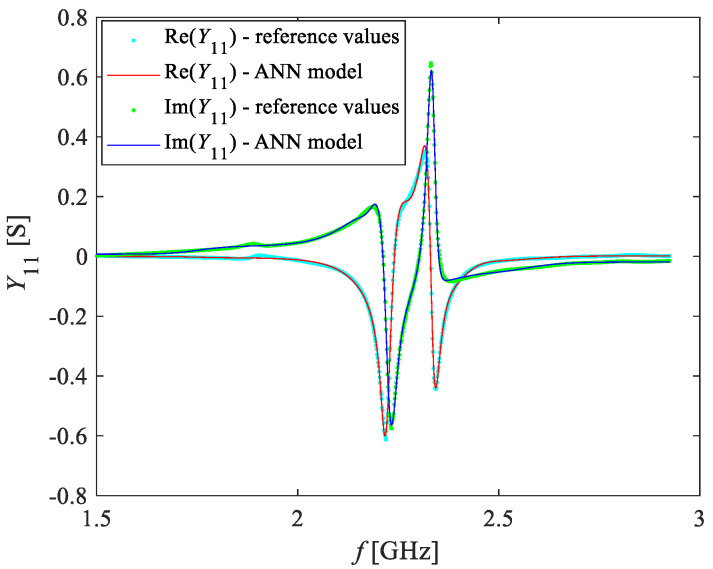
Frequency dependence of the real and imaginary parts of *Y*_11_ at 70% of the O_2_ concentration: (symbols) measurements and (lines) ANN simulations.

In the present case, the values of *Y*_11_, calculated from the measured *S*-parameters, were available at the following O_2_ concentrations: 0%, 5%, 10%, 15%, 20%, 40%, 70% and 100%. Upon analysis of the behavior of *Y*_11_ at these concentrations, 70% was selected as the reference concentration of O_2_. Therefore, ANN1 was trained with the data of *Y*_11_ referring to this concentration. The best ANN was the one with two hidden layers having five hidden neurons each. This ANN accurately mimics the real and imaginary parts of *Y*_11_, as can be seen in Figure 10.

**Figure 11:**
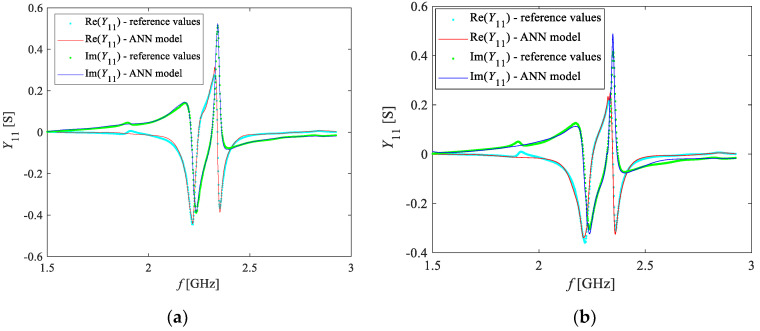
Frequency dependence of the real and imaginary parts of *Y*_11_ at two different values of the O_2_ concentration (symbols-measurements, lines-ANN simulations): (**a**) training concentration of 40%; (**b**) test concentration of 15%.

Afterwards, ANN2 was trained. From the available measurements, the data referring to the concentrations of 5% and 15% were left for the model verification, whereas the rest of the data was used for training purposes. As mentioned above, each measurement sample was accompanied by the corresponding values of *Y*_11_ that were calculated by using ANN1 for the same frequency, in order to be used as the ANN2 PKI inputs. After the training of several ANNs with a different number of hidden neurons, the ANN which gave the best compromise between the learning (i.e., the accuracy of predicting the training data) and the generalization (i.e., the accuracy of predicting the data not used for the ANN training) was chosen as the final model. This ANN model had two hidden layers consisting of 15 and 12 neurons, respectively. The ANNs were trained by applying the Levenberg–Marquardt training algorithm [56], which belongs to the backpropagation type of training algorithms. To illustrate the achieved learning and generalization accuracy, Figure 11 illustrates the real and imaginary parts of *Y*_11_ at different O_2_ concentrations. It can be noted that the values obtained by the ANN model (lines) closely match the measured values (symbols), not only for the training values of O_2_ concentration but also for the test values not used for the ANN training. This signifies that the ANN assimilated the training data and achieved a good generalization in predicting *Y*_11_ for the concentration values not considered during the model development.

**Figure 12:**
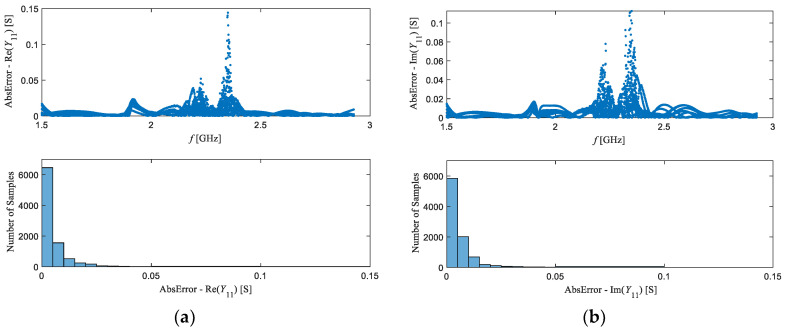
Frequency dependence and histograms showing the distribution of absolute values of the difference between the measured *Y*_11_ and the corresponding values obtained by using the ANN model: (**a**) *Y*_11_ real part; (**b**) *Y*_11_ imaginary part.

To quantify further the modelling accuracy, the absolute variation of the real and imaginary parts of *Y*_11_ simulated by the ANN model from the corresponding measured values is reported for all considered concentrations in Figure 12. Moreover, in Figure 12, the error distribution histograms are also reported. The histograms show how many of the considered total number of samples (i.e., the data points corresponding to different combinations of the frequency and the O_2_ concentration) have the value belonging to the range of each histogram bin. From these plots, one can see that the maximum absolute difference, for both real and imaginary parts, is smaller than 0.15 S, but in majority of the cases is even smaller than 0.05 S.

## 4. Results and Discussion

**Figure 13:**
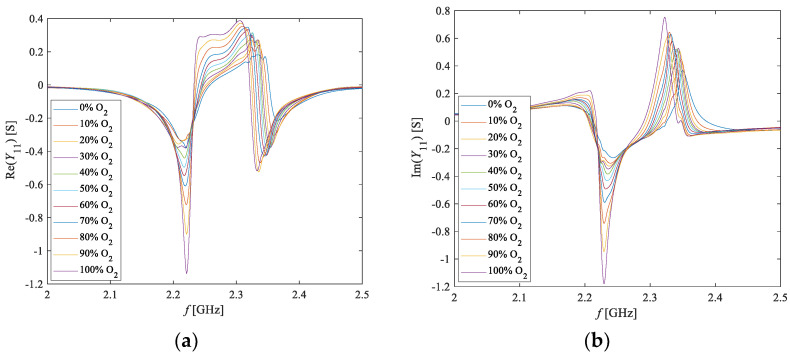
Frequency dependence of the (**a**) real and (**b**) imaginary parts of *Y*_11_ at different O_2_ concentrations, spanning from 0% to 100% with a step of 10%. These values are obtained by using the proposed ANN model.

The developed ANN model can be used to accurately predict the frequency-dependent behavior of *Y*_11_ for any O_2_ concentration value in a range of 0% to 100%. To illustrate this, Figure 13 shows the real and imaginary parts of *Y*_11_ at O_2_ concentrations with a step of 10%. The plots confirm that changes in the O_2_ concentration are clearly transduced into variations of *Y*_11_. In particular, there is a shift of the peaks and dips in the spectrum and there is also a change in their amplitude. At certain frequencies, the imaginary part of *Y*_11_ becomes null and thus this parameter is purely real. At these frequencies, the device resonates, and it is possible to relate the change in the resonant frequency to a change in the O_2_ concentration. Namely, three resonance frequencies can be identified, let them be named *f*_1_ (around 2.21 GHz), *f*_2_ (around 2.33 GHz), and *f*_3_ (around 2.38 GHz).

**Figure 14:**
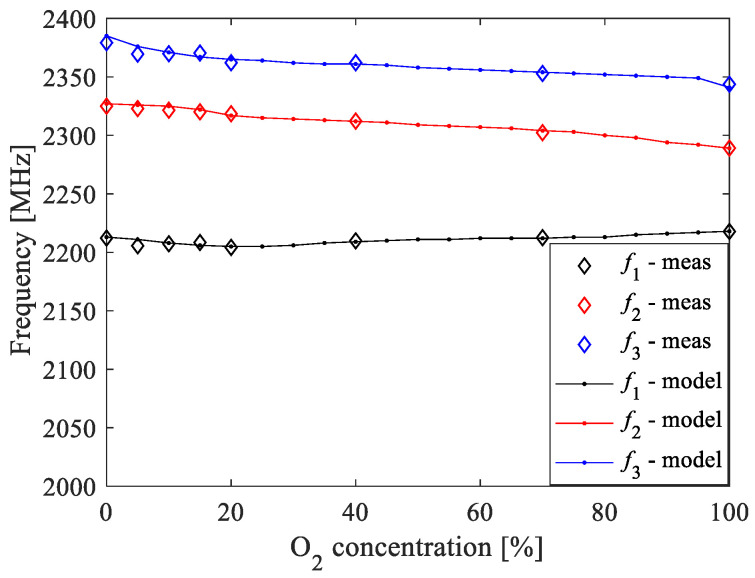
Comparison of the resonant frequencies determined by using the measured *Y*_11_ and the corresponding simulations based on the proposed ANN model.

Based on the analysis of the imaginary part of *Y*_11_ at different O_2_ concentrations, the resonant frequencies were read from the measurements and from the ANN model simulations, which were performed with a step of 5%. The obtained results are reported in Figure 14. It can be seen that the values obtained from the simulations agree very well with the values obtained from the measured *Y*_11_ for all considered concentrations. This demonstrates the accuracy of the developed ANN model and its generalization capability, enabling prediction of the resonant frequencies even at O_2_ concentrations different than the ones used during the experimental characterization.

**Figure 15:**
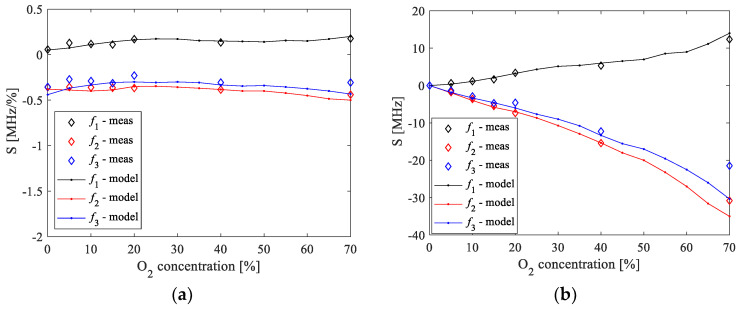
Comparison between measurements (symbols) and ANN simulations (lines) of the (**a**) absolute-absolute sensitivity and (**b**) absolute-relative sensitivity of the resonant frequencies to variations in the O_2_ concentration. The used reference value of the O_2_ concentration is 100%.

**Figure 16:**
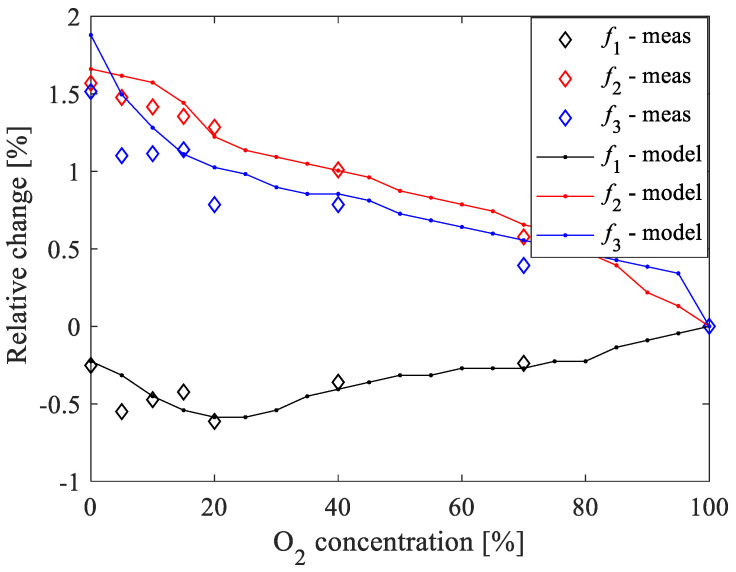
Comparison between measurements (symbols) and ANN simulations (lines) of the relative changes of the resonant frequencies versus the O_2_ concentration. The used reference value of the O_2_ concentration is 100%.

To investigate the suitability of the resonant frequencies as sensing parameters of the O_2_ concentrations, the sensitivity and the relative change versus the O_2_ concentrations are investigated. Figure 15 illustrates the absolute-absolute sensitivity and absolute-relative sensitivity of the resonant frequencies to changes in the O_2_ concentration by using 100% of O_2_ concentration as the reference value. The absolute-absolute sensitivity is calculated as $S=\left(f_{rx}-f_{r100}\right)/\left(\%O_{2}-100\right)$, whereas the relative-absolute sensitivity is calculated as $S=\%O_{2}\cdot \left(f_{rx}-f_{r100}\right)/\left(\%O_{2}-100\right)$. $f_{rx}$ is the considered resonant frequency at a certain $\%O_{2}$ and $f_{r100}$ is the corresponding resonant frequency at 100% of O_2_ concentration. For the sake of completeness, the relative change of the resonant frequencies, expressed as a percentage, is calculated as $100\cdot \left(f_{rx}-f_{r100}\right)/f_{r100}$ and the achieved results are given in Figure 16. It should be highlighted that *f*_1_ exhibits positive values for both sensitivities, whereas *f_2_* and *f_3_* have negative sensitivities. Figure 15a shows that the absolute-absolute sensitivity is roughly insensitive to the concentration of O_2_ for all of the three resonant frequencies. On the other hand, Figure 15b shows that the absolute values of the relative-absolute sensitivity increase linearly with the concentration of O_2_ for all of the three resonant frequencies. Although the sensitivities of the three resonant frequencies show similar behavior, the highest absolute values are achieved when using *f*_2_ and *f*_3_. (see Figure 15a,b). Therefore, these two resonant frequencies allow achieving an enhanced sensitivity of the proposed sensor for oxygen sensing. As the discrepancies between measured and simulated data are slightly larger when using *f*_3_, it can be concluded that *f*_2_ is the resonant frequency to be chosen as the sensing parameter in the present case.

## 5. Conclusions

A gas sensor has been developed and experimentally characterized, focusing on oxygen sensing as a case study. An ANN modelling approach has been proposed and successfully validated, enabling a faithful reproduction of the measured sensor performance and prediction even at oxygen concentrations not considered during the characterization phase. The assessment of the sensor sensitivity to variations in the oxygen concentration has been accomplished by analyzing the resonant frequencies, which were modelled versus the oxygen concentration by using the developed ANN model. Finally, it should be underlined that the achieved experimental and modelling results can find many practical sensing applications in the healthcare and bioengineering fields.
